# Prevalence of plasma lipid disorders with an emphasis on LDL cholesterol in selected countries in the Asia-Pacific region

**DOI:** 10.1186/s12944-021-01450-8

**Published:** 2021-04-15

**Authors:** Zhen-Vin Lee, Elmer Jasper Llanes, Renan Sukmawan, Nuntakorn Thongtang, Huynh Quang Tri Ho, Philip Barter, on behalf of the Cardiovascular RISk Prevention (CRISP) in Asia Network

**Affiliations:** 1 University Malaya Medical Centre, Kuala Lumpur, Malaysia; 2 Division of Cardiovascular Medicine, University of the Philippines, Manila, Philippines; 3 Department of Cardiology & Vascular Medicine, Universitas Indonesia, National Cardiovascular Center Harapan Kita, Jakarta, Indonesia; 4 Division of Endocrinology and Metabolism, Faculty of Medicine, Siriraj Hospital Mahidol University, Bangkok, Thailand; 5 Heart Institute, Ho Chi Minh City, Vietnam; 6 School of Medical Sciences, University of New South Wales, Sydney, NSW Australia

**Keywords:** Asia-Pacific, Dyslipidemia, Guidelines, Plasma lipid disorders, Prevalence

## Abstract

Cardiovascular disease (CVD) is a major cause of mortality and morbidity within the Asia-Pacific region, with the prevalence of CVD risk factors such as plasma lipid disorders increasing in many Asian countries. As members of the Cardiovascular RISk Prevention (CRISP) in Asia network, the authors have focused on plasma lipid disorders in the six countries within which they have clinical experience: Indonesia, Malaysia, Philippines, Thailand, Vietnam, and Australia. Based on country-specific national surveys, the prevalence of abnormal levels of total cholesterol, low- and high-density lipoprotein cholesterol (LDL-C and HDL-C, respectively), and triglycerides (TG) are reported. An important caveat is that countries have used different thresholds to define plasma lipid disorders, making direct comparisons difficult. The prevalence of abnormal lipid levels was as follows: high total cholesterol (30.2–47.7%, thresholds: 190–213 mg/dL); high LDL-C (33.2–47.5%; thresholds: 130–135 mg/dL); low/abnormal HDL-C (22.9–72.0%; thresholds: 39–50 mg/dL); and high/abnormal TG (13.9–38.7%; thresholds: 150–177 mg/dL). Similarities and differences between country-specific guidelines for the management of plasma lipid disorders are highlighted. Based on the authors’ clinical experience, some of the possible reasons for suboptimal management of plasma lipid disorders in each country are described. Issues common to several countries include physician reluctance to prescribe high-dose and/or high-intensity statins and poor understanding of disease, treatments, and side effects among patients. Treatment costs and geographical constraints have also hampered disease management in Indonesia and the Philippines. Understanding the factors governing the prevalence of plasma lipid disorders helps enhance strategies to reduce the burden of CVD in the Asia-Pacific region.

## Introduction

**Fig. 1:**
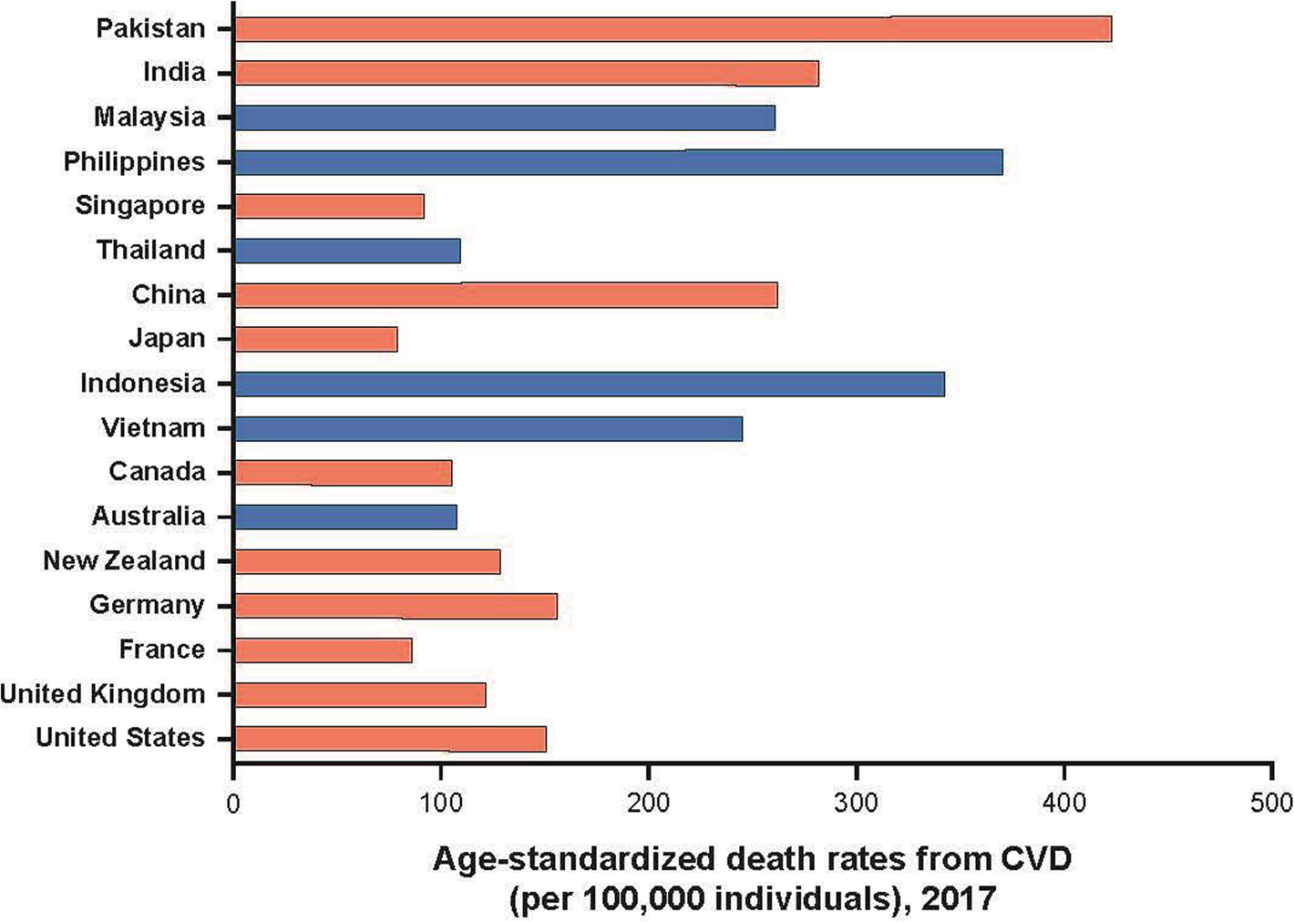
Age-standardized death rates per 100,000 from CVD [3]. Data from countries of interest are represented as black bars. Abbreviation: *CVD* cardiovascular disease

Cardiovascular disease (CVD) is one of the most prevalent and debilitating chronic diseases, and approximately half of the global burden of CVD is located in the Asia-Pacific region [1]. Further, the burden of CVD and plasma lipid disorders is increasing across the Asia-Pacific region [1, 2]. Age-standardized death rates per 100,000 for CVD in several Asian countries are among the highest in the world (Fig. 1). Further, the age-adjusted mortality due to cerebrovascular disease (stroke) in many Asian countries is higher than in some Western countries [1]. While age-specific CVD is declining in many high-income countries in other parts of the world, this is not the case in most of Asia [2, 4, 5].

**Fig. 2:**
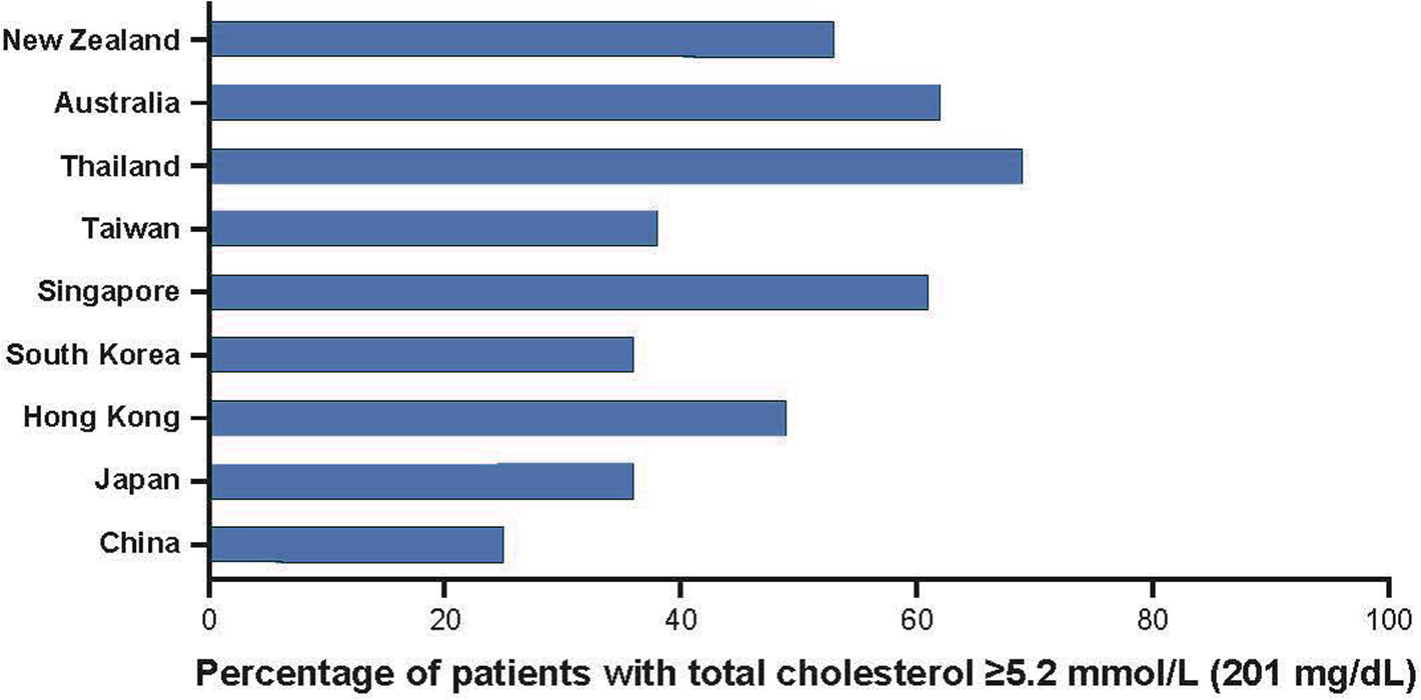
Percentage of patients with elevated total cholesterol in Asia and Australia/New Zealand [6]*. *Only studies from the Asia-Pacific Cohort Studies Collaboration that meet pre-defined criteria were used to generate these data [6]. The prevalence of plasma lipid disorders based on national survey data from Indonesia, Malaysia, Philippines, Thailand, Vietnam, and Australia is presented in Table 1

The prevalence of specific risk factors for CVD among Asian populations differs from Western populations in the same geographical region, such as Australia and New Zealand [6]. High blood pressure and high rates of smoking appear to be more prevalent in Asia whereas high plasma cholesterol (Fig. 2) and high body mass index (BMI) are more prevalent in Australia and New Zealand. However, the overall prevalence of cardiovascular (CV) risk factors is increasing across Asia, including plasma lipid disorders, obesity, and type 2 diabetes mellitus [7, 8]. A recent analysis by the NCD Risk Factor Collaboration suggested that from 1980 to 2018, the countries with the highest levels of non-HDL cholesterol had shifted from being countries in western Europe to those in Asia and the Pacific (including Malaysia, the Philippines, and Thailand) [9].

The Cardiovascular RISk Prevention (CRISP) in Asia network was convened in 2018 to gather clinical experts from different countries in the Asia-Pacific region. By sharing clinical insights and expertise across countries, the CRISP in Asia network aims to develop strategies to tackle the rising burden of CVD in the region. As members of the CRISP in Asia network, the authors’ aim for this review is to highlight the prevalence of plasma lipid disorders in selected Asian countries, with a specific focus on low-density lipoprotein cholesterol (LDL-C). In contrast to many previous studies, this review uses data from national surveys conducted in a cross-section of the general population, which the authors believe to be one of the best methods to obtain accurate estimates of the true prevalence of lipid disorders. The analysis concentrates primarily on the five countries in which the authors live and practice (Indonesia, Malaysia, Philippines, Thailand, and Vietnam), with Australia serving as a useful reference, being a representative Western country in the same region. As medical practitioners in these six countries, the authors are able to draw upon their clinical experience with patients with plasma lipid disorders. The review also describes country-specific guidelines that have been developed to reduce the prevalence and burden of plasma lipid disorders as well as the issues hampering these efforts. This review is not intended to be a set of guidelines but rather serves as a snapshot of the current state of plasma lipid disorders in selected Southeast Asian countries and Australia.

### Plasma lipid disorders

The term plasma lipid disorders, as used in this review, refers to abnormal levels of lipids in the blood, including LDL-C, high-density lipoprotein cholesterol (HDL-C), and triglycerides, which may lead to increased CV risk [10–12]. In Western populations, plasma lipid disorders typically refer to high LDL-C levels that are associated with increased CV risk. However, studies have shown an increased prevalence of low HDL-C and high triglyceride levels in Asian populations in comparison with Western populations, which may also be associated with increasing CV risk [5, 13, 14]. Treatment guidelines, which are often based on data from Western populations, typically focus on achieving low LDL-C and CV risk [15, 16]. Therefore, for the purposes of this review, the focus is predominantly on elevated LDL-C.

### Prevalence of plasma lipid disorders in selected countries in the Asia-Pacific region

Several studies evaluating the prevalence and management of plasma lipid disorders in the Asia-Pacific region have been conducted in recent years [17–33]. Many of these studies focus on specific subsets of a population, sampling only urban residents [17], or workers [22] or patients who are already receiving treatment for dyslipidemia [23], which may not reflect the prevalence within the general population of a particular country. A substantial proportion of people with dyslipidemia may not even be aware of their condition. Therefore, a more accurate estimate of the true prevalence can be gauged from surveys in which lipid levels are monitored in a cross-section of the general population. For this reason, the authors chose to use data from the most recent national surveys conducted in each country (Table 1); these surveys were usually undertaken by government agencies. Compared with specialized studies of prevalence, national surveys often have a larger sample size, cover a range of geographical areas (urban and rural), and sample both healthy and affected individuals.

**Table 1:** Prevalence of plasma lipid disorders across five Southeast Asian countries and Australia

Prevalence	Australia^**a**^	Indonesia^**b**^	Malaysia^**c**^	Philippines^**d**^	Thailand^**e**^	Vietnam^**f**^
Data source	Australian Health Survey: Biomedical Results for Chronic Diseases, 2011–12 [34]	Basic Health Research 2013 (Indonesia) [35]	National Health & Morbidity Survey (Malaysia) [36]	8th National Nutrition Survey Clinical and Health Survey (Philippines) [37]	National Health Examination Survey V (Thailand) [38]	National Survey on the Risk Factors of Non-Communicable Diseases (STEPS) Viet Nam, 2015 [39]
Definition of high or ‘abnormal’ total cholesterol	≥5.5 mmol/L (213 mg/dL)	≥200 mg/dL	≥200 mg/dL	≥200 mg/dL	≥200 mg/dL	≥ 5.0 mmol/L or ≥ 190 mg/dL or currently on medication for elevated cholesterol
Prevalence of high or ‘abnormal’ total cholesterol (%)	32.8	35.9	47.7	47.2	43.8	30.2
Male vs. female (%)	32.4 vs. 33.2	30.0 vs. 39.6	43.5 vs. 52.2	41.9 vs. 51.8	40.8 vs. 46.7	25.2 vs. 35.0
Urban vs. rural (%)	–	39.5 vs. 32.1	47.7 vs. 47.7	50.7 vs. 43.5	45.2 vs. 42.7	–
Age group with highest prevalence (years)	55–64	–	55–59	50–59	45–59	50–69
Definition of high or ‘abnormal’ LDL-C	≥3.5 mmol/L (135 mg/dL)	≥130 mg/dL	–	≥130 mg/dL	–	–
Prevalence of high LDL-C (%)	33.2	41.9	–	47.5	–	–
Definition of low or ‘abnormal’ HDL-C	< 1.0 mmol/L for males (39 mg/dL) and < 1.3 mmol/L for females (50.3 mg/dL)	< 40 mg/dL	–	< 40 mg/dL for males and < 50 mg/dL for females	< 40 mg/dL for males and < 50 mg/dL for females	< 1.03 mmol/L or < 40 mg/dL; HDL-C for females: < 1.29 mmol/L or < 50 mg/dL
Prevalence of low or ‘abnormal’ HDL-C (%)	23.1	22.9	–	71.0	40.3	67.0 (males) 72.0 (females)
Definition of high or ‘abnormal’ TG	≥2.0 mmol/L (177.14 mg/dL)	≥150 mg/dL	–	≥150 mg/dL	≥150 mg/dL	–
Prevalence of high or ‘abnormal’ TG (%)	13.9	24.9	–	38.7	31.0	–
Sample size	~ 14,000	~ 39,000	~ 16,000	~ 19,000	~ 19,000	~ 4000
Sampling method	Stratified multistage area sampling of private dwellings	Multistage systematic random area sampling of households	Two stage stratified random area sampling of households	Multistage stratified area sampling of households	Stratified multistage sampling of population	Multistage stratified area sampling of households
Urban and/or rural sampling	Both urban and rural (very remote areas excluded)	Both urban and rural	Both urban and rural	Both urban and rural	Both urban and rural	Both urban and rural
Age range	≥18 years old	≥15 years old	≥18 years old	≥20 years old	≥15 years old	18–69 years old
Year data acquired	2011–2012	2013	2015	2013–2014	2014	2015
Finger prick	No	No (10 mL)	Yes	No (venous)	No (venous)	Yes
Fasted	Yes	–	Yes	Yes	Yes	–

In instances where thresholds were provided in mmol/L, the equivalent threshold has also been expressed as mg/dL, which has been calculated using previously published conversion factors [40]

*Abbreviations*: *HDL-C* high-density lipoprotein cholesterol, *LDL-C* low-density lipoprotein cholesterol, *TG* triglycerides

National survey data for each of the six countries above were provided by the authors or were obtained by web searches of the publicly available resources for the relevant organizations in each country (e.g., Australian Bureau of Statistics, Ministry of Health of Republic of Indonesia, Ministry of Health: General Department of Preventative Medicine [Vietnam]). In order to be included in this publication, the survey data had to satisfy the following criteria: (1) data had to have been collected within the last 10 years by a government agency, (2) sampling had to be performed on the general population (instead of a focus on patients with a confirmed diagnosis) in both urban and rural areas, (3) data had to contain information on the prevalence of high/abnormal total cholesterol, as well as other relevant lipid parameters. The relevant data were extracted (categories were translated to English if required) and tabulated as shown in Table 1. Data on familial hypercholesterolemia were not present in surveys from every country so were not included in the present analysis.

It is important to note that because some countries have used different thresholds to define plasma lipid disorders, it is difficult to compare the prevalence of these disorders across countries. As such, we have reported the prevalence of lipid disorders for each country (where available) but have refrained from making comparisons between countries. At least 30% of participants sampled in all six countries had high total cholesterol (thresholds range 190–213 mg/dL) (Table 1). The prevalence of high total cholesterol ranged from 30.2% (Vietnam) to 47.7% (Malaysia). Among the three countries with data collected for LDL-C, the prevalence of high LDL-C was 33.2% for Australia, 41.9% for Indonesia, and 47.5% for the Philippines (Table 1); all three countries employed similar thresholds to define high LDL-C. The five countries that collected data on the prevalence of low HDL-C were Australia (23.1%), Indonesia (22.9%), the Philippines (71%), Thailand (40.3%), and Vietnam [67% (males); 72% (females)]. The prevalence of high triglycerides was reported for Australia (13.9%), Indonesia (24.9%), the Philippines (38.7%), and Thailand (31.0%).

A number of countries also examined the differences in the prevalence of lipid disorders among different subgroups. In all six countries, the prevalence of high total cholesterol was numerically higher in females compared with males (Table 1), with the largest differences (~ 9%) observed in Indonesia, the Philippines, and Vietnam. Prevalence also appeared to be highest in the age brackets between 50 and 69 years, for Australia, Malaysia, the Philippines, and Vietnam (Table 1). In Thailand, the 45–59-year age group had the highest prevalence of high total cholesterol. In terms of socio-economic status, the prevalence of borderline to high total cholesterol in the Philippines was observed to increase with the wealth of respondents (poorest quintile: 33.0% vs richest quintile: 57.8%); a similar trend was observed for the prevalence of LDL-C. In Malaysia, the prevalence of high total cholesterol was similar across all household income quintiles (46.0–52.2%).

Although the data reported for all six countries come from national surveys, there were important differences in the way that the surveys were carried out, which may affect the estimated prevalence. For example, Lin and coauthors described several factors that can affect the validity of comparing study findings, including the age range of participants, lipid testing methods, medications used, year of data acquisition, and definition of dyslipidemia used for each study [23].

Data from all six countries were collected during a similar period (2011–2015) and were from both urban and rural populations (Table 1). The survey sample sizes were similar for Australia, Malaysia, the Philippines, and Thailand (between 14,000 and 19,000), while a much larger sample was surveyed in Indonesia (~ 39,000) and a much smaller sample surveyed in Vietnam (~ 4000). The minimum age of participants was between 18 and 20 years for Australia, Malaysia, the Philippines, and Vietnam, while participants in Indonesia and Thailand had lower minimum ages (≥15 years old). There were notable differences in blood sampling methods across countries, with finger prick sampling employed in Malaysia and Vietnam but not in the other countries, which mostly used venous sampling.

### Guidelines for improving the management of plasma lipid disorders

Given the prevalence of plasma lipid disorders in Asian and Western countries, several international guidelines have been developed to reduce CV risk; these guidelines are based on robust evidence, largely from randomized controlled clinical trials. Clinical practice guidelines to reduce CV risk are available from international committees such as the European Society of Cardiology (ESC) and European Atherosclerosis Society (EAS) [16] and the recently published 2018 AHA/ACC/AACVPR/AAPA/ABC/ACPM/ADA/AGS/APhA/ASPC/NLA/PCNA guidelines [15]. These major international guidelines served as the basis for the development of country-specific guidelines. Indonesia [41], Malaysia [42], the Philippines [43], Thailand [44], and Vietnam [45] have all developed local guidelines (Table 2). However, given that the development of these international guidelines was based mainly on clinical trial data from Western populations, their applicability to Asian countries needs to be confirmed.

**Table 2:** Local guidelines for management of plasma lipid disorders

	CV risk scoring system used	Goal	Recommended treatments for		
			Primary prevention	Patients with diabetes	Secondary prevention
Indonesia (*Indonesian Heart Association Guidelines on Management of Dyslipidemia 2017*) [41]	• Stratification of CV risks as low, medium, high, or very high • SCORE scale is most commonly used • Jakarta Cardiovascular score (Modified Framingham Risk) has also been introduced based on local data [46]	• Very high risk: LDL-C < 70 mg/dL and/or 50% reduction if baseline 70–135 mg/dL • High risk: LDL-C < 100 mg/dL or 50% reduction if baseline 100–200 mg/dL • Moderate risk LDL-C < 115 mg/dL	• Lifestyle intervention includes diet, physical activity, BMI reduction, and smoking cessation • Statins may be initiated with lifestyle intervention for those with high risk and very high risk	• Similar strategy with very high risk and high risk categories • For those with ASCVD or target organ damage, LDL-C goal < 70 mg/dL; for those without ASCVD or target organ damage, LDL-C < 100 mg/dL • If target LDL-C cannot be reached with highest tolerated doses of statin, non-statin therapy may be considered	• Statins for all patients unless statin intolerant • Additional non-statin therapy with ezetimibe or PCSK9 inhibitors if LDL-C goals not achieved with highest tolerated dose of statins
Malaysia (*Management of Dyslipidemia 2017*) [42]	• CV risk scores (Framingham General CVD) used	• Low and intermediate CV risk: < 3.0 mmol/L (116 mg/dL) • High CV risk: ≤2.6 mmol/L (100 mg/dL) or a reduction of > 50% from baseline • Very high CV risk: < 1.8 mmol/L (70 mg/dL) or a reduction of > 50% from baseline	• TLC recommended Statins for those with high and very high CV risk as well as those with low and moderate CV risk after TLC	• Statins for all patients with diabetes > 40 years • High-intensity statins for patients with diabetes and CVD	• High-intensity statins for all patients with CHD or ACS and prior to PCI and CABG
Philippines (*2015 Clinical Practice Guidelines for the Management of Dyslipidemia in the* *Philippines*) [43]	• Risk factor counting to identify patients in need of statins	• < 130 mg/dL (or a 30% reduction) for those at lower risk • < 70 mg/dL (or > 30% reduction) for those with established ASCVD	• Statins for non-diabetic patients aged ≥45 years with LDL-C ≥ 130 mg/dL and ≥ 2 risk factors without atherosclerotic CVD and for diabetic individuals without atherosclerotic CVD	Regardless of age of CV risk, guidelines recommend initiation of moderate- intensity statin therapy	• High-intensity statin (based on LDL-C reduction)
Thailand (*2016 RCPT Clinical Practice Guideline on Pharmacologic Therapy of Dyslipidemia for Atherosclerotic Cardiovascular Disease Prevention*) [44]	• CV risk score used based on Thai patient population (Thai CV Risk Score)	• < 130 mg/dL (or a 30% reduction) for those with 10-year risk ≥10% • < 100 mg/dL for primary prevention in DM, CKD, or familial hypercholesterolemia • < 70 mg/dL (or a 50% reduction) for clinical ASCVD	• Statins for patients with LDL-C ≥ 190 mg/dL, familial hypercholesterolemia, 10-year risk ≥10%	• Statins for patients: - DM ≥ 40 years - DM < 40 years + 2 CV risk factors + LDL-C ≥ 100 mg/dL (moderate intensity statins) - DM < 40 years with 0 or 1 CV risk factor + LDL-C ≥ 100 mg/dl (low to moderate intensity statins)	• Statins (moderate to high intensity depending on atherosclerotic CVD) • Non-statin if LDL-C target not reached in 6 months
Vietnam (*Recommendations on Diagnosis and Treatment of Lipid Disorders 2015*)	• SCORE scale (low risk) used • Stratification of CV risk as low, medium, high, or very high	ESC recommendations for goals Primary < 100 mg/dL Secondary < 70 mg/dL	Statins for patients with LDL-C ≥ 190 mg/dL	• Statins for patients aged 40–75 with diabetes and LDL-C between 70 and 189 mg/dL • High-intensity statins used for most patients with diabetes	• Statins for patients with atherosclerosis, acute coronary syndrome, history of MI, stable/unstable angina • LDL < 70 mg/dL

Abbreviations: *ACS* acute coronary syndrome; *ASCVD* atherosclerotic cardiovascular disease; *CABG* coronary artery bypass grafting; *CHD* coronary heart disease; *CKD* chronic kidney disease; *CV* cardiovascular; *CVD* cardiovascular disease; *DM* diabetes mellitus; *eGFR* estimated glomerular filtration rate; *HbA1c* glycated hemoglobin; *LDL-C* low-density lipoprotein cholesterol; *MI* myocardial infarction; *PCI* percutaneous coronary intervention; *TLC* therapeutic lifestyle changes

In addition to country-specific or regional guidelines, there are also expert panel recommendations on the use of international guidelines in Asia [5]. A 2018 expert panel (which included Philip Barter and Nuntakorn Thongtang) noted that the 2016 ESC/EAS guidelines have introduced specific LDL-C goals for different risk groups and promote lifestyle intervention (considered generally applicable to the management of plasma lipid disorders in Asia-Pacific). The 2014 National Institute for Health and Care Excellence UK guidelines [47] were considered by the panel to be applicable in Malaysia, the Philippines, Thailand, and Indonesia.

The expert panel concluded that LDL-C is an important CV risk factor, with the failure to attain optimal lipid levels contributing significantly to the residual risk of CVD and that therefore, LDL-C should be a primary therapeutic target. The panel also recommended that there should be a major emphasis on lifestyle intervention, whether or not drug therapy is used, and the decision to use lipid-lowering drugs should be based on an assessment of overall CV risk rather than on any perceived need to treat an abnormal lipid level. Lastly, the panel also recognized the potential for less intensive statin therapy to be used in the Asian population [5] and recommended that local guidelines be developed that are appropriate for the local populations [5, 48].

However, since these panel recommendations were published, both major international guidelines have been updated, with the 2018 AHA/ACC and the 2019 ESC/EAS guidelines being the most recent. The panel did not consider the Vietnamese guidelines nor the most recent update to the Indonesian guidelines, which occurred in 2017. Compared with the 2013 AHA/ACC guidelines, the 2018 AHA/ACC guidelines recommended the addition of ezetimibe for those receiving maximal statin therapy with LDL-C still ≥70 mg/dL [15]. If needed, PCSK9 inhibitors were recommended for patients with very high clinical atherosclerotic CVD risk. Similarly, the revised 2019 ESC/EAS guidelines recommended the addition of ezetimibe when LDL-C goal is not achieved with the maximum tolerated dose of statin. In terms of secondary prevention, the addition of a PCSK9 inhibitor was recommended for very high-risk patients not at goal despite treatment with the maximum tolerated dose of statins and ezetimibe.

### Comparison of local guidelines for management of plasma lipid disorders

The use of major international guidelines as a basis for the development of country-specific guidelines has resulted in a number of similarities between the guidelines of the Asian countries being discussed. Almost all countries recommended the use of a risk scoring system to estimate CV risk (Table 2). The exception was the Philippines, which recommended risk factor counting due to concerns that CV risk calculators based on Western patients were not validated in the Filipino population. Similar concerns were raised in Malaysia, where a recent study examined the validity of different risk-prediction models, concluding that the Framingham Risk Score and the SCORE-high risk models could be used in the Malaysian population [49]. The SCORE-low risk model is used to estimate the 10-year risk of fatal CVD in Vietnam [45]. Unlike most of the other countries, the CV risk score used in Thailand has been validated in a local Thai patient population [50]. All countries had specific LDL-C goals that depended on an individual’s level of CV risk (Table 2). Most countries recommend LDL-C < 70 mg/dL for those at highest CV risk and LDL-C < 116 to 130 mg/dL for those at low to moderate CV risk.

In terms of primary prevention, all five countries (Table 1) recommended statins, although guidelines in Malaysia, Indonesia, and the Philippines also advocated CVD prevention through healthy lifestyle changes. Malaysian primary prevention strategies also involved identifying individuals who are likely to develop CVD and obtaining lipid profiles for them, in addition to formulating dietary and lifestyle recommendations specifically tailored for Malaysian patients [42]. For secondary prevention, most countries recommended the use of high-intensity statins, with the Thai guidelines recommending non-statins if the LDL-C goal was not achieved within 6 months. Unlike the most recent AHA/ACC and ESC/EAS guidance, few local guidelines recommended the use of PCSK9 inhibitors.

It should be noted that updated guidelines for the management of plasma lipid disorders are currently not available for Australia and thus have not been included in Table 2. Most Australian clinicians use either the US or European Union guidelines.

### Studies of lipid-lowering treatments conducted in the Asia-Pacific region

Lipid-lowering treatments are an essential component of the guidelines for each of the countries being discussed. In order to assess the effectiveness of lipid-lowering treatments and lipid goal attainment by patients, a number of major international studies have been initiated. Several of these studies also collated data from countries in the Asia-Pacific region; these studies include CEPHEUS (pan-regional study of hypercholesterolemia) [51], DYSIS (hyperlipidemia in the setting of chronic statin treatment), DYSIS II (hyperlipidemia in patients with acute and stable CHD) [52], and PRIMULA (mixed plasma lipid disorders) [53].

**Fig. 3:**
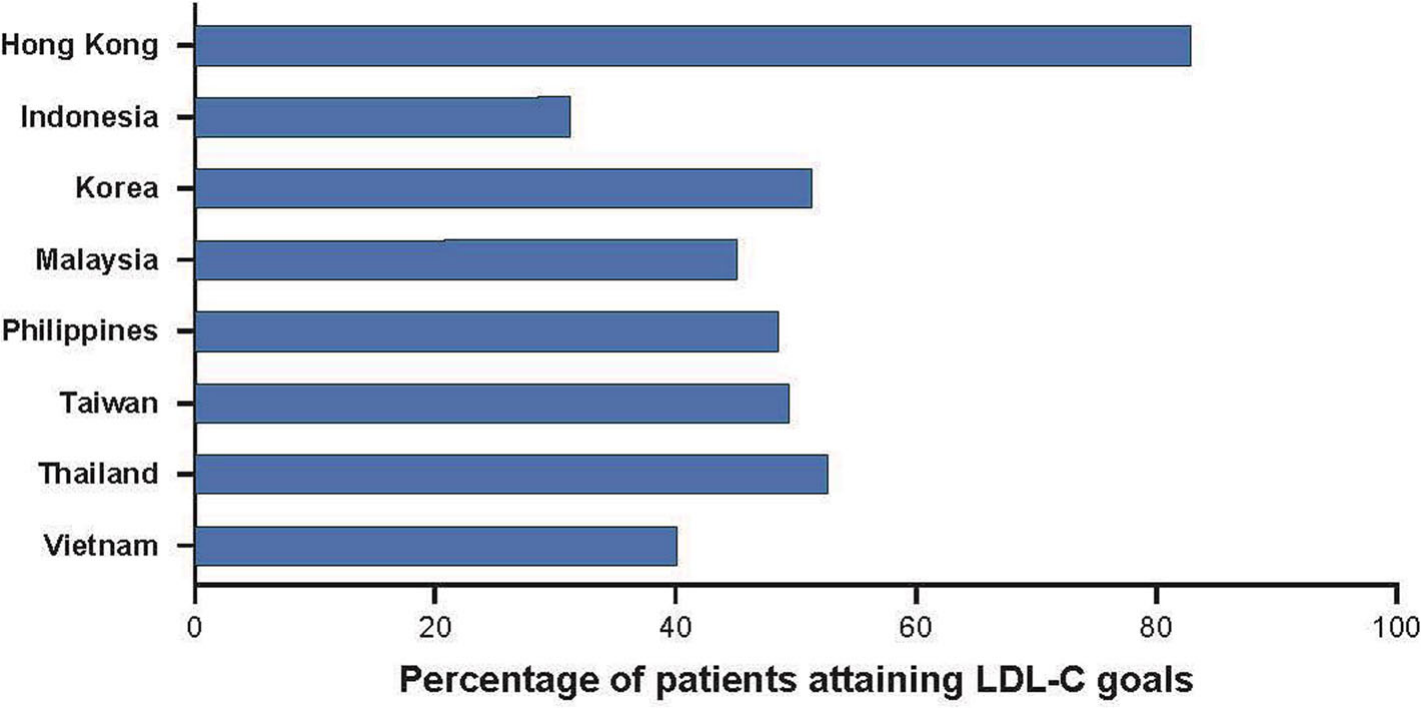
Percentage of patients attaining LDL-C goals in Asian countries within the CEPHEUS study [51]. Abbreviation: *LDL-C* low-density lipoprotein cholesterol

In CEPHEUS, CV risk category and attainment goals for LDL-C were assessed according to the updated NCEP ATP III guidelines, which were in use when the study was conducted (moderately high risk: LDL-C < 130 mg/dL; high risk: LDL-C < 100 mg/dL; very high risk: LDL-C < 70 mg/dL). Among patients who were receiving lipid-lowering treatments in 29 countries, approximately half (49.4%) achieved their recommended LDL-C level (54.8 and 22.8% of patients at high or very high CV risk, respectively) [54]. The Asian patients (Fig. 3) included in CEPHEUS demonstrated similar levels of goal attainment (49.1%) compared with patients from all 29 countries [51]; however, there were substantial differences in goal attainment across the Asian countries, with Hong Kong showing the highest proportion of patients at goal, at 82.9% (Fig. 3).

The attainment of LDL-C goals in Indonesia (31.3%) [55] was below that of the overall rate in Asia (49.1%), with the lowest attainment (12.1%) found in patients with a therapeutic target of < 70 mg/dL. Goal attainment was inversely related to CV risk and baseline LDL-C (*P* < 0.001). In Thailand [56], 52.7% of patients reached their LDL goal (16.7% in the very high risk group, 60.6% in the high risk group, and 84.7% in the moderately high risk group).

Variations in goal attainment between the Asian countries were thought to result from several factors, including differences in practice patterns and medication pricing [51]. The authors also reported that several factors were significantly associated with LDL-C goal attainment, including medication used, patient age, blood pressure, total cholesterol, and LDL-C levels at baseline, therapeutic LDL-C goals used, and compliance with treatment.

**Fig. 4:**
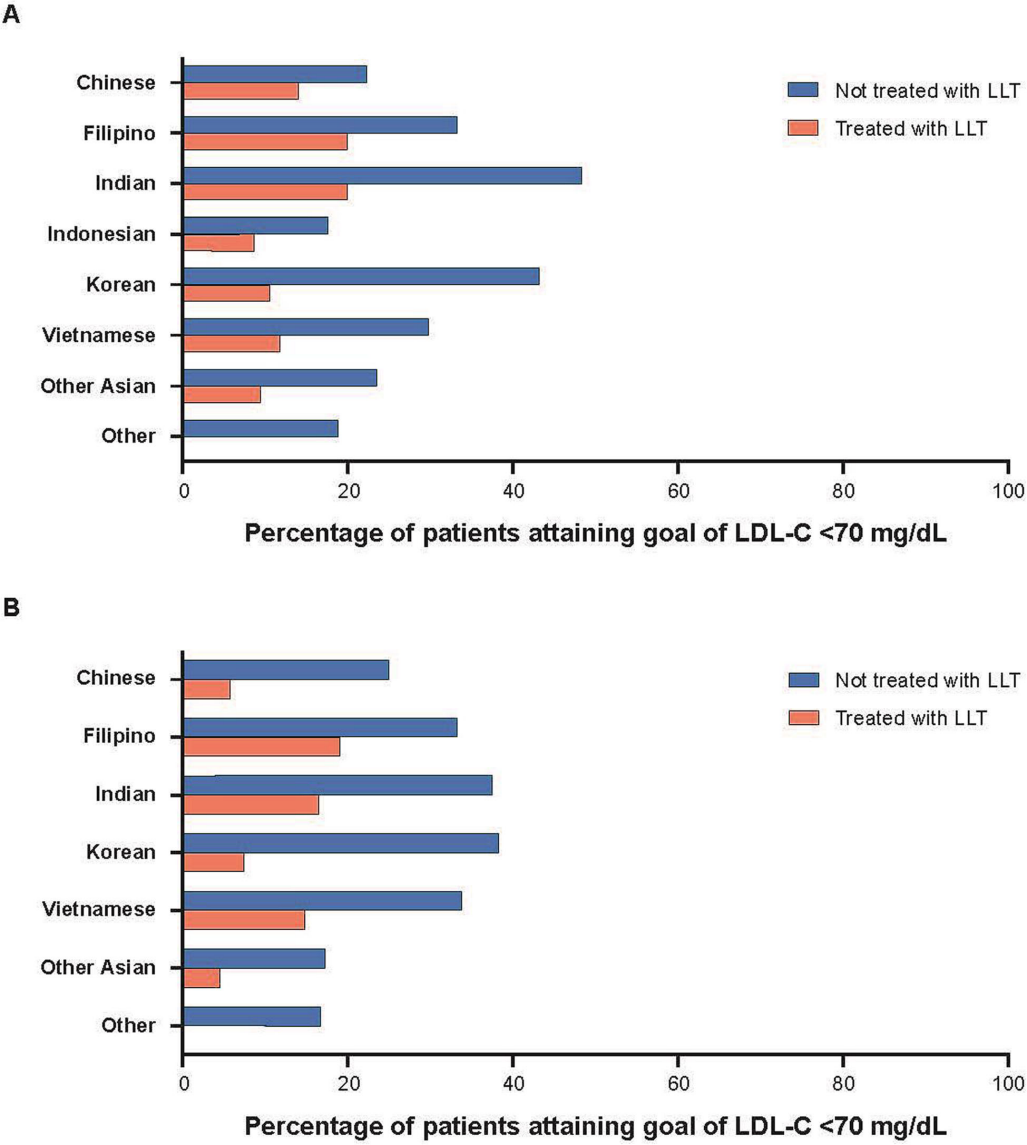
Goal attainment (LDL-C < 70 mg/dL) for Asian patients with **a** coronary heart disease and **b** acute coronary syndrome in the DYSIS-II study [52]. Abbreviations: *LDL-C* low-density lipoprotein cholesterol; *LLT* lipid-lowering therapies

In DYSIS, CV risk category and attainment goals were based on ESC/EAS 2011 guidelines (moderate risk: LDL-C < 115 mg/dL; high risk: LDL-C < 100 mg/dL; very high risk: LDL-C < 70 mg/dL). Only 21.7% of very high risk statin-treated patients attained < 70 mg/dL LDL-C [57]. In DYSIS II, CV risk category and attainment goals were based on ESC/EAS 2011 guidelines for very high risk patients (LDL-C < 70 mg/dL). Many patients across Asia at very high risk of CV events had an LDL-C level above the recommended target [52]. In patients with highest risk, those with manifest acute coronary syndrome, reduction of LDL-C remained low (41.7%). The proportions of patients in Hong Kong, India, Indonesia, the Philippines, Singapore, South Korea, Taiwan, Thailand, and Vietnam attaining LDL-C < 70 mg/dL are shown in Fig. 4 [52].

In PRIMULA, CV risk category and attainment goals for LDL-C were assessed according to the updated NCEP ATP III guidelines (moderately high risk: LDL-C < 130 mg/dL; high risk: LDL-C < 100 mg/dL; very high risk: LDL-C < 70 mg/dL). A pooled analysis was conducted across Malaysia, Korea, Hong Kong, Thailand, and the Philippines [53]. Lipid-modifying therapy reduced the prevalence of plasma lipid disorders, but a third of patients still failed to achieve target/normal levels [53]. Patient characteristics associated with attaining LDL-C goals included patient risk (higher probability for non-smokers, non-diabetics, and non-CVD), gender (higher probability for men), and country (higher probability in Korea, lower probability in Malaysia and the Philippines). In Thailand, after 12 months of lipid-modifying therapy, 21% of patients overall (26% in high risk group) still failed to attain LDL-C goals [58]. Lower LDL-C level at baseline was a significant predictor of goal attainment.

A real-world study in Thailand reported that the proportion of patients not at LDL-C goal was 77.7% for patients with established atherosclerotic disease (goal: < 70 mg/dL) and 43.0% for patients with multiple risk factors [59].

Based on the studies above, there is a disconnect between the proportion of patients with known plasma lipid disorders, those treated, and those whose lipid levels are controlled. Although the use of lipid-lowering therapy is common across the Asia-Pacific region, it is not used to its full potential, especially in very high risk patients [52, 60]. In order to develop more effective management strategies, a better understanding of the reasons for poor goal attainment is needed.

### Barriers to lipid goal attainment

Based on the clinical experience of the authors, there are several barriers to the attainment of recommended lipid goals, with a number of obstacles common across countries. There appears to be a reluctance amongst physicians in the Philippines, Malaysia, Thailand [61, 62], and Vietnam to use high-dose and/or high-intensity statins, even for secondary prevention. The use of suboptimal doses of statins was well-characterized in several Asian countries in the DYSIS-II study [52]. Despite guideline recommendations, some physicians in Vietnam are also reluctant to prescribe high-dose statins due to concerns about side effects. In the Philippines, some physicians in rural areas lack awareness of the latest clinical guidelines and/or trials and are thus less confident in prescribing higher-dose statins to patients. In Malaysia, suboptimal secondary prevention in patients with pre-existing coronary artery disease has been partly ascribed to poor physician prescribing patterns and patient compliance [60]. High workloads for GPs and primary care physicians in Malaysia can lead to rushed consultations, making the formation of partnerships between patients and physicians for the management of CV risk difficult. Furthermore, the timing of follow-up visits is not standardized and is often 6–9 months after the first consultation.

Patient attitudes to disease and treatment may also be barriers to lipid goal attainment. In Malaysia and Vietnam, patients are concerned about the potential side effects of statins, as many are influenced by the wealth of information (and misinformation) on the internet and on social media. Some patients do not appreciate the importance of controlling hypercholesterolemia as the condition may not result in any appreciable symptoms. Patients in the Philippines, particularly those in rural areas, are often reluctant to seek medical care unless they develop symptoms. Instead, some patients prefer to use herbal supplements, which are often recommended as a cure-all by friends, relatives, or social media. Patients in Malaysia also had doubts about the efficacy of lipid-lowering treatments as they did not provide immediate symptomatic relief, in contrast to other types of medications, such as analgesics. However, doubts concerning efficacy may not apply to all treatments; recent single-center studies in Italy suggest that the use of PCSK9 inhibitors may be associated with improved quality of life (as reported by patients) [63] and adherence to treatment [64]. It would be interesting to see if these findings also hold true for patients in the Asia-Pacific region.

The geographical make-up of the Philippines and Indonesia, with their populations spread across thousands of islands, poses unique challenges from a healthcare perspective. The management of CV risk and plasma lipid disorders can vary considerably from one area to another due to unequal distribution of healthcare resources. Populations in rural areas (e.g., mountainous areas and islands) face difficulties in accessing healthcare facilities, treatment, and healthcare workers. All these factors contribute to the low attainment of LDL-C goals in rural areas.

Until recently, healthcare funding has also been an issue in both Indonesia and the Philippines. However, the implementation of universal health coverage in Indonesia in 2014 [65] and the recent signing of the Universal Healthcare Act into law in the Philippines may ease some of the financial constraints for patients who would otherwise have to pay out of their own pocket (especially for medications for chronic conditions, such as diabetes, hypertension, and plasma lipid disorders in the Philippines). Despite the implementation of universal health coverage in most countries, the availability of some treatments may still be limited. In Indonesia, universal health coverage covers the cost of low- and moderate-intensity statins but not high-intensity statins. Therefore, patients who are prescribed high-intensity statins may be required to make out-of-pocket payments. This can result in physicians being reluctant to prescribe high-intensity statins or patients being unable to access statins of the appropriate intensity, leading to lower goal attainment. The low utilization of high-intensity statins observed for several countries in this region is likely due to multiple factors. However, based on the data compiled for this review, it is difficult to definitively identify the factors specific to each country. As such, further research is needed to explore the reasons behind this important observation.

Although countries like Thailand have had universal health coverage for almost 20 years, the percentage of the population with high total serum cholesterol who are effectively treated remains small. Many of those affected are unaware of their condition, as reported in a 2004 study showing that 78% of affected individuals in Thailand were undiagnosed [66]. Although the proportion of undiagnosed patients in Thailand decreased in the following 10 years, 61.7% still remained undiagnosed according to the 2014 national health examination survey [38]. The underdiagnosis of high total cholesterol can be attributed to the general Thai population’s lack of awareness about plasma lipid disorders or a failure to appreciate the importance of disease screening for the primary prevention of atherosclerotic CVD.

### Strengths and limitations

The key strength of this review is the fact that it draws upon national survey data from each country of interest. Rather than focus on a specific subset of the population, national surveys generate data from a cross-section of the general population, capturing both affected and healthy individuals. For this reason, national survey data may provide a more accurate estimate of the true prevalence of lipid disorders in the general population, compared with specialized studies of prevalence. Another strength of the study is the country-specific, clinical experience of the authors, which provides valuable insights into the logistical, cultural, and regulatory issues faced by patients in each country. The main limitations of this review are the differences in the survey methods used in each country and the different thresholds used to categorize lipid disorders, which make direct comparisons between countries difficult. Nevertheless, the data presented provide a valuable snapshot of the current state of lipid disorders in the Asia-Pacific region.

## Conclusions

Despite the widespread availability of both efficacious and well-tolerated lipid-lowering therapies and treatment guidelines, the prevalence of plasma lipid disorders is rising in the Asia-Pacific region. The clinical and economic impact of such an increase is likely to be substantial, given the age-adjusted mortality due to CVD being higher in many Asian countries than in Western countries. In the first of a planned series of publications focused on plasma lipid disorders in the Asia-Pacific region, this review highlights the prevalence of these disorders and defined several barriers to lipid goal attainment. Underdiagnoses, undertreatment, and suboptimal management of plasma lipid disorders is seen throughout the region, leading to poor goal attainment, even among those at high risk of CV events. In the future, improved approaches (as more information becomes available) need to be applied across the entire management continuum. Such approaches include improving screening for and diagnosis of plasma lipid disorders, increasing patient awareness and access to medications, and optimizing the use of lipid-lowering treatments. Improving adherence to therapeutic lifestyle changes and lipid-lowering therapy and the intensification of treatment to ensure that patients are treated to their appropriate LDL-C goal are also recommended for this region. In a future publication, the authors hope to expand upon some of these approaches in greater detail and discuss their possible impact.

## Data Availability

The datasets supporting the conclusions of this article are included within the article.

## Abbreviations

ACS: Acute coronary syndrome
ASCVD: Atherosclerotic cardiovascular disease
BMI: Body mass index
CABG: Coronary artery bypass grafting
CHD: Coronary heart disease
CKD: Chronic kidney disease
CV: Cardiovascular
CVD: Cardiovascular disease
DM: Diabetes mellitus
eGFR: estimated glomerular filtration rate
HbA1c: Glycated hemoglobin
HDL-C: High-density lipoprotein cholesterol
LDL-C: Low-density lipoprotein cholesterol
LLT: Lipid-lowering therapies
MI: Myocardial infarction
PCI: Percutaneous coronary intervention
TC: Total cholesterol
TG: Triglycerides
TLC: Therapeutic lifestyle changes
